# The impact of the COVID‐19 pandemic on the health and economy of Nepal

**DOI:** 10.1002/puh2.85

**Published:** 2023-05-04

**Authors:** Srista Manandhar, Sunit Chhetri, Swapnil Regmi, Sunny Chhetri, Thinley Dorji

**Affiliations:** ^1^ Department of Internal Medicine B. P. Koirala Institute of Health Sciences Dharan Nepal; ^2^ Department of Internal Medicine Central Regional Referral Hospital, Ministry of Health Gelegphu Bhutan

**Keywords:** food security, health services, Himalayas, Nepal, pandemic, school education

## Abstract

Nepal experienced a devastating earthquake in 2015, sweeping political changes and restructuring of its healthcare system in the last decade. The economy is heavily dependent on tourism with an average of 1 million visitors per year prior to the pandemic. The country had just begun the ‘Visit Nepal 2020’ campaign when the government had to realign its economic activities and allocate resources to battling the impacts of the COVID‐19 pandemic. The tourism sector crashed with 18.7% of the population falling into poverty. The health system faced reeling pressure with interruptions of routine services while the government struggled to provide care to COVID‐19 patients. There was a shortage of 20,000 healthcare workers and derailment of important health services, such as antenatal care, institutional delivery of babies and tuberculosis diagnosis. To fund the COVID‐19 vaccination efforts, the government sought donations and loans from other countries. There were disruptions in schools and the education system which had challenges in the delivery of education through digital means due to limited internet services. The agriculture and the food production system also faced major disruptions leading to exorbitant inflation and an increase in the cost of living. The country opened to normal socio‐economic activities by May 2022 in an effort to reverse the impacts on various sectors of life in Nepal. We describe how Nepal, a resource‐constrained country handled the COVID‐19 pandemic.

## INTRODUCTION

The COVID‐19 pandemic had devastating social and economic impacts on low‐ and middle‐income countries with a disproportionate burden on vulnerable sections of the population. Nepal is one such country with a population of 29,192,480 and a gross domestic product of 33.65 billion US dollars that suffered from the unprecedented impacts of COVID‐19 [1, 2].

As the pandemic unfolded, the government adopted social distancing measures and nationwide lockdowns, including the sealing of international borders with neighbouring countries, India and China, and halting of air travel. Nepal had multiple cycles of outbreaks with a total of 252,740 cases and 12,019 deaths reported from across the country as of 1 November 2022 [3]. Some of the major travel restrictions and nationwide lockdowns were imposed during March–September 2020 [4] and April–September 2021 [5]. This brought about the major sectors of the economy and social functions to a standstill. We discuss the impacts of the COVID‐19 pandemic on various sectors of Nepalese and Nepali lifestyles.

## HEALTH

Nepal has a government‐run healthcare system restructured into local, provincial and state‐run facilities since 2015 with Primary Health Care Centre (PHC), Basic Health Centres and Health Posts as basic units for health service delivery. Access to healthcare is a challenge in many parts of Nepal due to geographic, economic or social factors. Nepal also faces a shortage of health workers where the doctor/patient ratio stands at 0.8 per 1000 population, one of the lowest in the region [6].

Nepal took multiple early actions to combat the pandemic, imposed nationwide lockdown, made prompt adaptive changes in national policies and regulations across all sectors, allocated a national budget to combat COVID‐19 and sought out multiple loan deals from the World Bank and Asian Development Bank. By mid‐2021, Nepal had invested Nepalese Rupees 23.16 billion (US dollar 174 million) on COVID‐19 prevention and control measures with the majority being spent on building infrastructure [7]. By 2021, the number of healthcare facilities in the government sector increased by 2% compared to 2020 with a count reaching up to 201 hospitals, 189 Primary Healthcare Centres and 3794 Health Posts [8]. A total of 22,127 quarantine beds, 13,700 isolation beds, 1154 intensive care unit beds, 475 ventilators and 676 high dependency units were added by mid‐2021 [7]. By 2021, as the COVID‐19 surge increased, the government had received a total of 4126 oxygen concentrators, 6945 oxygen cylinders, 1.48 million oxygen kits, 13,820 body bags and 218 mechanical ventilators through various domestic and international governmental and non‐governmental aids [9]. In December 2020, Nepal laid the foundation for the construction of 300 hospitals, each with a capacity of 5–15 beds, to be completed in 2 years' time [7]. By May 2022, there were 78 government‐based COVID‐19 hospitals with a total of 2797 critical care beds (1.6% in Kathmandu Valley) and 106 reverse transcriptase polymerase chain reaction testing labs [3]. Government efforts in constructing physical infrastructures were commendable but hiring new health human resources and training already existing resources was lacklustre.

The limited human resources, scarce personal protective equipment and tiring working hours were notable challenges. The World Health Organization has estimated that 180,000 healthcare workers had lost their lives by May 2021 due to COVID‐19 [10]. In Nepal, many healthcare and frontline workers lost their lives, but the actual data reflecting the true numbers are unavailable. There was a shortage of 20,000 healthcare workers [11], whereas the number of healthcare workers declined by 0.6% from 90,946 in July 2020 to 90,369 in March 2021. Lumbini Province, despite best attempts, could only hire 463 health personnel of the required 583.

In addition to the unavailability of adequate health personnel, the delivery of routine health services was disrupted. With the surge of COVID‐19 cases, nationwide lockdowns and travel restrictions prevented people from visiting health facilities which was further aggravated by the fear and stigma of visiting healthcare facilities during the time of the pandemic. Additionally, the conversion of many healthcare centres to COVID‐19 hospitals hampered services for chronic health‐related conditions and non‐communicable diseases. Tuberculosis diagnosis was reduced to 50% possibly due to the use of face masks or a reduction in diagnostic tests [12].

Patients were deprived of regular antenatal care visits, preconception counselling, family planning visits and neonatal services. The weekly institutional birth rate decreased by 52.4% from January to May 2020 which previously was 651.4 births per week. Similarly, institutional stillbirth increased from 14 to 21 per 1000 births and institutional neonatal mortality increased from 13 to 40 per 1000 live births during the same period. Additionally, women from disadvantaged communities were deprived of access to institutional healthcare facilities. For example, the proportion of people seeking healthcare services in the Madhesi community fell to 17.1% from 21.5% in December 2019 [13]. Mental health–related issues were on the rise across the country. The suicide rate increased by 0.28 per 100,000 population since the beginning of the lockdown in July 2020 compared to 2017–2019 [14]. There were notable increases in the cases of anxiety, depression and insomnia [15].

With all these hurdles in providing healthcare services during the pandemic, the use of digital communication aided the patients in accessing key essential services and health information [16]. Telemedicine, the digital health consultation, was a convenient solution, particularly during the travel restrictions during pandemic. It has also been a cost‐effective means for patients to receive consultations, particularly for COVID‐19‐related symptoms and follow‐ups for chronic illnesses such as hypertension and diabetes. The availability of quality digital health services in rural areas reduced the need for referral if the problems were addressed promptly at the local level [17]. The continuation of telemedicine services post‐lockdown has benefitted in having continued access to healthcare services.

Regarding the COVID‐19 vaccination campaign in Nepal, donations of vaccines by neighbouring countries provided a major boost to initiate the campaign. Further donations and investment by the government helped further sustain the campaign. By the end of May 2022, 76.8% of the population had been vaccinated with at least a single dose, 68.06% has been vaccinated with both doses and 14.03% has been vaccinated with booster doses. Vaccines registered in Nepal include AstraZeneca, Sinopharm VeroCell, Pfizer, Johnson & Johnson, Moderna and Sinovac‐CoronaVac vaccine.

## TOURISM

Tourism is an important source of income for Nepal with an average of 1 million visitors per year prior to the pandemic. The most popular attractions of Nepal include Mount Everest, Lumbini – the birthplace of Gautam Buddha, Pokhara and the Annapurna region. With the start of the pandemic, the global tourism industry came to a crash, including that of Nepal with the halt to the ‘Visit Nepal 2020’ campaign and an 80% reduction in the number of tourists compared to 2019 and 70% reduction in foreign currency income [18]. Travel and tourism contributed 4.3% of GDP in 2020 and 2021 and 7.5% in 2019. Nepal reported an overall unemployment rate of 2.85% with 18.7% of the population living in poverty (per capita income less than 1.9 US dollars per day) [19, 20]. The number of jobs (porters, mountain and trekking guides, hotels, restaurants and travel agencies) in the tourism industry fell to 1.03 million in 2020 and 1.06 million in 2021 compared to 1.16 million in 2019 leading to job loss among 11.6% of the population in this sector [21]. Foreign currency remittance has not changed during the pandemic, with remittance contributing 22.5% to the overall GDP during the fiscal year 2019/20 and 2020/21. The contribution of remittance to the overall GDP of 2022 is not available yet, but the percentage change of remittance till mid‐March of 2022 was −1.7% underlining the overall decrease [22]. However, in the last 5 months of 2022, remittances had increased by 23% compared to the same period of the previous year, underlining the overall improvement in conditions [23]. By May 2022, the pandemic restrictions had come to an end, and the country has re‐opened its border to international travel and tourism [24].

## AGRICULTURE AND FOOD PRODUCTION

Nepal is a land of mountains, hills and the Terai region along its southern border. The unique weather and the cultivable plain land marks Nepal as an agricultural country contributing to 25.8% of GDP in the year 2020/2021 and involving 66% of the population in farming [25]. The pandemic had major disruptions in the production, import and export of livestock and food. The arable area for food production has increased by 0.5% in the fiscal year 2020/21 due to the return of people to their native villages during the lockdown. Nepal imports a substantial quantity of food (meat, fish, cardamom, ginger, chilly and fruits) [18] from India and China and exports the raw materials to those countries. The closure of international borders led to the restriction of the import of commodities, including food items leading to inflation and increasing costs of living. The long periods of lockdown limited factories from operating, farmers from working in the field and in transporting perishable food items such as milk and vegetables for sale. A higher burden of food scarcity was reported among the lower income bracket leading to increasing cases of childhood malnutrition and infectious diseases [15].

According to a World Bank assessment, 32% went jobless in the farming sector among which 40% were waged workers [26]. Unlike other sectors, because of the seasonality of cultivation and crop production, the recovery of the agricultural sector is likely to lag behind the other industries contributing to economic growth.

## EDUCATION

Nepal has 35,055 schools, 70% of which are community‐based public schools. There are 1432 higher‐level institutions with 441,819 students. After COVID‐19‐related travel restrictions, lockdowns and implementation of social distancing, academic activities came to a pause for a long duration with drastic changes in the academic calendar. Many of the school premises were converted into quarantine centres for COVID‐19 cases.

In Nepal, internet facilities are available to only 12% of schools and 56% of households [27]. Some villages do not have internet facilities even now. Even for those who had access, internet subscription was an additional expenditure. The other challenges were a lack of infrastructure, logistics and trained educators for the delivery of effective digital education [15, 28]. Hence, the transition of the delivery of education during the pandemic was a delicate task with Nepal lacking in overall infrastructure and trained human resources.

## LESSONS LEARNT

The mega‐earthquake in 2015 and the COVID‐19 pandemic provided many lessons on the management of crises and disasters in a resource‐constrained setting like Nepal. The government has taken steps to boost significant production in the agricultural sector, incentivize migrant workers to retain them and diversify sources of economic income. The government has also taken efforts in improving the access to healthcare services, technology and internet in providing telemedicine and school education. Training of relevant human resources has been initiated to provide virtual education to children in rural areas [27].

Emerging and re‐emerging diseases will continue to pose challenges from time to time. There must be an expansion of health research capacity, training and workshops in strengthening the capability of outbreak investigation and control in the future, including improvements in testing capability, test–trace–isolate infectious cases and availability of treatment facilities, such as intensive care units, mechanical ventilators and trained infectious disease specialists. It is equally important to motivate and incentivize health workers working in high‐risk situations such as the COVID‐19 pandemic. The healthcare system must find the means and capability to provide uninterrupted routine care services in women and reproductive health, mental health, non‐communicable diseases, road traffic accidents and victims of physical, mental or sexual violence [29].

The pandemic has demonstrated how the federal system of governance can bring together local multisectoral stakeholders in formulating proper strategic plans and policies, conducting awareness and enhancing preparedness at the grassroot level, responding to local epidemiology of disease outbreaks and handling local health budgets [30].

Learning from the disruption in the supply chains of key consumables, masks and personal protective equipment, Nepal has collaborated with the private sector and initiated the production of health items for both domestic consumption and international supply.

## CONCLUSION

The COVID‐19 pandemic was a major challenge to the socio‐economic and health sectors in Nepal. It has demonstrated an urgent need for capacity building and improvement in system resilience in the health sector to manage pandemics or health disasters in the future, manage the delivery of routine essential health services, improve food security, provide education to children and maintain political harmony in the federal structure of government. It is equally important not to overlook the major sectors of the country's economy and support the small businesses, farmers and tourism sectors.

## AUTHOR CONTRIBUTIONS

All authors were involved in the conception of this manuscript. Srista Manandhar and Sunit Chhetri drafted the manuscript. Swapnil Regmi and Sunny Chhetri assisted in collecting data. Thinley Dorji was involved in editing and reviewing the manuscript. All authors were involved in critical review and have given final approval for publication of the materials.

## CONFLICT OF INTEREST STATEMENT

Thinley Dorji is a board member of the editorial committee of this journal. Thinley Dorji was excluded and blinded from all stages of the peer review of this manuscript.

## ETHICS STATEMENT

Ethical clearance is not applicable to this manuscript.

## FUNDING

None

## Data Availability

All relevant data sources are cited in this article.
